# Distribution of malaria exposure in endemic countries in Africa considering country levels of effective treatment

**DOI:** 10.1186/s12936-015-0864-3

**Published:** 2015-10-05

**Authors:** Melissa A. Penny, Nicolas Maire, Caitlin A. Bever, Peter Pemberton-Ross, Olivier J. T. Briët, David L. Smith, Peter W. Gething, Thomas A. Smith

**Affiliations:** Department of Epidemiology and Public Health, Swiss Tropical and Public Health Institute, 4051 Basel, Switzerland; University of Basel, Petersplatz 1, Basel, Switzerland; Institute for Disease Modeling, Bellevue, WA 98005 USA; Department of Zoology, University of Oxford, Tinbergen Building, South Parks Road, Oxford, OX1 3PS UK; Sanaria Institute of Global Health and Tropical Medicine, Rockville, MD 20850 USA

**Keywords:** Malaria, Transmission, Case-management, Simulation, Dynamic model

## Abstract

**Background:**

Malaria prevalence, clinical incidence, treatment, and transmission rates are dynamically interrelated. Prevalence is often considered a measure of malaria transmission, but treatment of clinical malaria reduces prevalence, and consequently also infectiousness to the mosquito vector and onward transmission. The impact of the frequency of treatment on prevalence in a population is generally not considered. This can lead to potential underestimation of malaria exposure in settings with good health systems. Furthermore, these dynamical relationships between prevalence, treatment, and transmission have not generally been taken into account in estimates of burden.

**Methods:**

Using prevalence as an input, estimates of disease incidence and transmission [as the distribution of the entomological inoculation rate (EIR)] for *Plasmodium falciparum* have now been made for 43 countries in Africa using both empirical relationships (that do not allow for treatment) and OpenMalaria dynamic micro-simulation models (that explicitly include the effects of treatment). For each estimate, prevalence inputs were taken from geo-statistical models fitted for the year 2010 by the Malaria Atlas Project to all available observed prevalence data. National level estimates of the effectiveness of case management in treating clinical attacks were used as inputs to the estimation of both EIR and disease incidence by the dynamic models.

**Results and conclusions:**

When coverage of effective treatment is taken into account, higher country level estimates of average EIR and thus higher disease burden, are obtained for a given prevalence level, especially where access to treatment is high, and prevalence relatively low. These methods provide a unified framework for comparison of both the immediate and longer-term impacts of case management and of preventive interventions.

**Electronic supplementary material:**

The online version of this article (doi:10.1186/s12936-015-0864-3) contains supplementary material, which is available to authorized users.

## Background

**Fig. 1:**
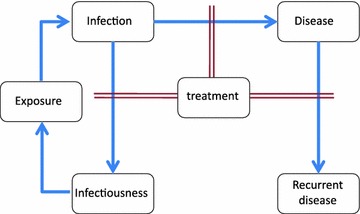
Illustration of impact of treatment effectiveness on interactions between infection, clinical disease and exposure. *Arrows* indicate causal links and *double lines* show where treatment has a modifying effect

The prevalence of *Plasmodium falciparum* infections is routinely measured in malaria indicator surveys (MIS), and as part of various health assessments and research projects. Prevalence data are therefore relatively widely available and are often used as a measure of endemicity in geographical comparisons and in evaluating the success of intervention programmes [1]. However, although prevalence is a consequence of malaria transmission and levels of exposure, these variables do not have a one-to-one relationship but rather a non-linear relationship modified by many factors such as naturally acquired immunity, malaria interventions and of heterogeneity in transmission rates [2]. These complicate the interpretation of age-patterns of infection and disease. The relationship between exposure and prevalence of infection also depends on the amount of treatment in the population because treatment truncates infections and (depending on the drug regimen) provides a few weeks of chemoprophylaxis (Fig. 1). If access to effective treatment is good, then prevalence may remain relatively low, even at high transmission levels. The amount of effective treatment also affects the relationships of exposure (or prevalence) with morbidity, and mortality rates (Fig. 1).

Human exposure to malaria, one part of malaria transmission, is best quantified by the entomological inoculation rate (EIR: the number of infectious bites per human host, per unit time), which is more directly related to morbidity and mortality than is prevalence. However, measuring this quantity directly requires intensive entomological studies over the whole annual period of malaria transmission. Previously established empirical relationships between prevalence and EIR have illustrated the complications and diversity by site [3]. EIR data are consequently relatively sparse, and indirect methods, that ideally account for treatment effects, are needed for estimating EIR from available prevalence data [4, 5].

The comprehensive repository of geo-located malaria parasite prevalence data maintained by the Malaria Atlas Project (MAP) is the obvious starting point for estimating how many people are exposed to malaria at different intensities, in different endemic countries. Several different algorithms have been used to infer the distribution of exposure from prevalence maps. In particular, a linear relationship between prevalence and the logarithm of the EIR approximates the empirical relationship between these variables [6], and the MAP repository includes EIR surfaces and estimates of the uncertainty based on this relationship [7]. Other researchers use process models to estimate transmission rates surfaces from prevalence data [6, 8–10]. These analyses do not allow for effects of treatment on prevalence. At low transmission levels, where infection events are sporadic, and superinfection relatively infrequent, this omission can be remedied using rather simple models for translating prevalence into transmission estimates, conditional on the incidence of effective treatment [4]. At higher levels of transmission, both concurrent and sequential superinfection are frequent; so mechanistic models allowing for this, as well as for treatment rates, are needed.

Estimates of the number of clinical malaria episodes at national level and continent-wide have been made from the MAP database by assuming a standard empirically determined relationship between prevalence and the incidence of clinical malaria in children [11, 12]. Using a similar methodology based on geographical stratification of risk, estimates of clinical incidence at national level are made yearly by the World Health Organization (WHO) for the World Malaria Report (WMR) for high-burden sub-Saharan countries [13]. This report also provides up-to-date assessments of malaria-related interventions and policies, attempting to quantify the impact on disease burden. Estimates of clinical incidence for each year have been made by adjusting for changing intervention coverage levels within each country, assuming effects match those seen in controlled trials [14].

These estimates of clinical incidence do not allow for levels of access to effective treatment. This affects both the true extent of pathology, and the observed clinical incidence, whether ascertained passively or actively. Depending on underlying exposure, high treatment levels create a virtuous cycle by averting further pathology and secondary cases. Estimates of worldwide and national levels of burden should, therefore, take into account effects of treatment, as well as the shifts in age patterns of prevalence [15] and of incidence that occur as a result of transmission reducing interventions.

The OpenMalaria platform supports an ensemble of models that can be used for calibrating different malariological indices against each other [16]. OpenMalaria is a stochastic, individual-based, simulation model of malaria in humans [17] linked to a deterministic model of malaria in mosquitoes [18]. The simulation model includes sub-models of infection of humans [19], blood-stage parasite densities [20], infectiousness to mosquitoes [21], incidence of morbidity including severe and hospitalisation [22, 23] and mortality [22]. An ensemble of 14 model variants is available [24] with each model including different assumptions for decay of natural immunity, greater within-host variability between infection and entomological exposure, heterogeneity in transmission and heterogeneity in susceptibility to co-morbidities.

Six of the OpenMalaria ensemble models were used in this work to compute estimates of the distribution of exposure (EIR) for each of 43 malaria endemic countries in sub-Saharan Africa as well as estimates of clinical incidence (and also incidence of severe disease and malaria mortality) for 2010 levels of malaria control. These estimates are based on the pixel-level posterior distributions of parasite prevalence in 2010 published by MAP [7]. For each country, these estimates are conditional on national level estimates of the levels of access to effective treatment for malaria fevers [25]. The resulting estimates of the distribution of transmission and of the incidence of clinical malaria provide a basis for evaluating the impacts of both preventive and curative intervention programmes allowing for the effects of existing case management on prevalence and burden of disease.

## Methods

An overview of the methods in estimating malaria exposure distributions (as EIR) and resulting burden is presented in Table 1, including inputs and outputs of each method.

**Table 1:** Description of the EIR and burden estimation methods A and B including their inputs and outputs

Method	Description	*Pf*PR_2–10_ (input by pixel)	Population demographics (input by pixel)	Coverage of clinical treatment	EIR (output and input to burden calculations)	Burden (output from OpenMalaria simulations)
A	Malaria transmission as EIR is estimated using previous published statistical relationship between prevalence and EIR [6, 7]. Burden of clinical disease is determined via the OpenMalaria micro-simulation model with EIR distributions derived by this method as inputs	Prevalence distributions from MAP [7] by pixel (5 km by 5 km). See Fig. 3a and results Additional file 2: Figure S2	Population numbers by pixel from [30]	Input for burden estimation only: coverage of effective treatment is country or geographic area specific (Table 2, [25])	Using the empirical relationship between prevalence and EIR [6, 7] (Eq. 1) and the prevalence distributions per pixel weighted by population demographics, a population weighted distribution for EIR is constructed. Overall EIR distribution for a geographic area is found by aggregation of the pixel EIR distributions (Fig. 3c and results Figs. 4, 5)	Using population weighted EIR distributions from Method A as input and assuming coverage of treatment at country specific levels, clinical incidence is determined using the OpenMalaria micro-simulation (process schematic Fig. 3d and results Fig. 7)
B Assuming country levels of coverage of effective treatment	Malaria transmission as EIR is estimated using a derived functional form of the relationship between prevalence and EIR and level of effective treatment from the OpenMalaria micro-simulation model. Burden of clinical disease is determined via the OpenMalaria microsimulation model with EIR distributions derived by this method as inputs	Prevalence distributions from MAP [7] by pixel (5 km by 5 km). Figure 3a and results Additional file 2: Figure S2	Population numbers by pixel from [30]	Input for both EIR and burden estimation: Coverage of effective treatment is country or geographic area specific (Table 2, [25])	A statistical relationship is fit to predict prevalences from the OpenMalaria micro-simulation model for a range of EIR and different levels of coverage of effective treatment of clinical disease (Fig. 3b). Prevalence distributions weighted by population by pixel are transformed to EIR distributions via this fitted function (Eq. 2) for country levels of coverage of effective treatment resulting in a population weighted distribution of EIRs by pixel and aggregated to country or geographic area. (Fig. 3c and results Figs. 4, 5).	Using population weighted EIR distributions from Method B as input and assuming coverage of treatment at country specific levels, clinical incidence is determined using the OpenMalaria micro-simulation (process schematic Fig. 3d and results Fig. 7)
B Assuming coverage of effective treatment at pre-ACT scale up levels	As above	As above	As above	Input for both EIR and burden estimation: E_14_ = 15 % capturing the situation before recent scale-up of ACT	As above but assuming coverage of effective treatment is 15 %	As above but EIR distributions and incidence are determined assuming coverage of effective treatment is 15 %

### Malaria prevalence data

National levels of prevalence were taken from the prevalence surfaces estimated by the Malaria Atlas Project (MAP) for *Plasmodium falciparium* 2010 [7]. Estimates of prevalence in children aged 2 up to children before their 10th birthday (*Pf*PR_2–10_) across a 5 km × 5 km grid were extracted as posterior distributions from a Markov Chain Monte Carlo (MCMC) calculated via a Bayesian geostatistical model using survey data. The primary estimates are of *Pf*PR_2–10_ are available from the MAP website [26] as posterior densities.

### National levels of effective treatment coverage

National levels of access to effective malaria treatment were collated previously [25] and are detailed in Table 2. Effective malaria treatment is treatment that results in parasitological cure. In this work effective treatment are estimates of the probability, *E*_14_, that effective malaria treatment will be obtained during any 14-day period in which a fever occurs. Estimates were assembled at country level taking into account multiple factors for effectiveness of malaria case management, including probably of treatment-seeking, of type of care provider, of systems compliance with the recommended anti-malarial treatment, of adherence with the drug regimen, and the quality of the anti-malarial medications.

**Table 2:** Coverages of effective treatment and estimated transmission profiles (EIR mean, median and quartiles) for 43 sub-Saharan Africa estimated by method B assuming country level effective treatment

Country	Country code	Coverage of effective treatment [25] (E_14_ %)	Weighted mean EIR	Weighted median EIR	Weighted EIR 25 % quartile	Weighted EIR 75 % quartile
Angola	ago	48.7	49.8	6.2	1.4	50.4
Benin	ben	30.3	72.8	13.3	2.9	130.3
Botswana	bwa	71.3	5.3	0.0	0.0	1.7
Burkina Faso	bfa	34.6	118.1	73.7	9.1	407.3
Burundi	bdi	42.6	13.5	1.1	0.2	6.2
Cameroon	cmr	25.9	67.4	11.0	2.0	107.8
Central African Republic	caf	13.4	61.5	7.6	1.1	89.2
Chad	tcd	17.7	27.8	1.7	0.2	11.0
Comoros	com	37.6	46.5	5.2	0.8	34.5
Congo	cog	42.9	49.0	7.6	1.7	50.4
Democratic Republic of Congo	cod	26.9	47.4	4.3	0.6	34.5
Cote d’Ivoire	civ	25.3	78.8	19.5	2.9	157.6
Djibouti	dji	46.6	0.2	0.0	0.0	0.0
Equatorial Guinea	gnq	19.4	76.8	16.1	2.4	157.6
Eritrea	eri	24.7	1.1	0.1	0.0	0.2
Ethiopia	eth	15.8	1.0	0.0	0.0	0.1
Gabon	gab	40.4	71.7	16.1	3.5	130.3
The Gambia	gmb	39.3	7.3	1.4	0.4	5.2
Ghana	gha	39.9	52.3	7.6	1.7	50.4
Guinea	gin	25.0	39.7	3.5	0.6	23.6
Guinea Bissau	gnb	27.5	6.3	0.8	0.2	2.9
Kenya	ken	35.7	7.7	0.2	0.0	1.1
Liberia	lbr	45.2	60.2	16.1	4.3	73.7
Madagascar	mdg	20.2	42.0	2.0	0.2	23.6
Malawi	mwi	39.1	54.5	9.1	1.7	61.0
Mali	mli	27.5	76.0	16.1	2.4	157.6
Mauritania	mrt	22.4	5.4	0.1	0.0	0.4
Mozambique	moz	37.9	65.8	11.0	1.7	107.8
Namibia	nam	38.0	11.3	0.3	0.0	2.4
Niger	ner	30.9	35.3	3.5	0.8	19.5
Nigeria	nga	32.2	65.7	11.0	2.4	107.8
Rwanda	rwa	41.0	2.2	0.2	0.0	0.9
Sao Tome Principe	stp	68.0	25.8	6.2	1.7	23.6
Senegal	sen	32.3	5.8	0.6	0.2	2.4
Sierra Leone	sle	36.8	61.0	11.0	2.4	73.7
Somalia	som	7.5	1.1	0.0	0.0	0.2
North Sudan	sdn	18.8	7.0	0.1	0.0	0.5
South Sudan	ssd	8.7	17.0	0.1	0.0	2.0
Tanzania	tza	44.5	25.1	2.0	0.4	11.0
Togo	tgo	18.1	58.9	7.6	1.4	73.7
Uganda	uga	66.3	89.7	34.5	7.6	190.6
Zambia	zmb	51.5	26.9	2.9	0.6	16.1
Zimbabwe	zwe	25.7	2.8	0.2	0.1	0.6

### Relationship between parasite prevalence and EIR

Two different methods were used to estimate distributions of malaria exposure (as measured by the entomological inoculation rate, EIR) from *Pf*PR_2–10_ data:

#### Method A: statistical relationship between EIR and prevalence

**Fig. 2:**
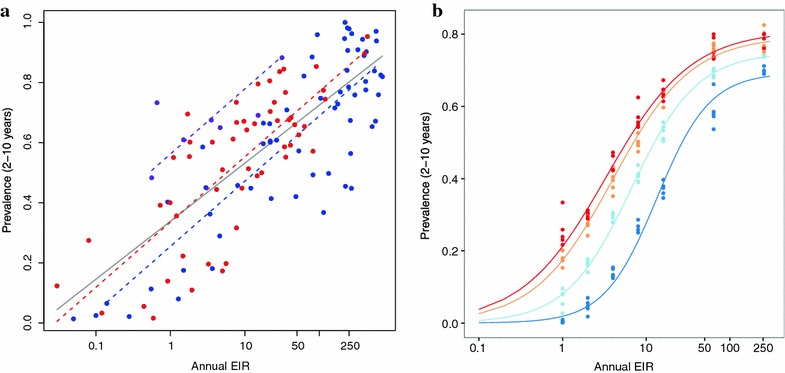
Relationships between malaria exposure (EIR), effective coverage, and prevalence for Method A (**a**) and Method B (**b**). **a** Method A: plotted empirical relationship of prevalence as a function of EIR relationship [6] (Eq. 1) with data used to fit this relationship. The relationship between standardized prevalence and EIR is approximately linear-log for all the data (*grey curve* fitted relationship over all data). The relationship varies by study (*purple colour* *dots* a correspond to data from a single field study, *purple curve* the fitted relationship to those data) and by method (*red dots* correspond to measurements taken via pyrethroid spray catches, and *purple* and *blue dots* measurements taken by other methods. *Red* and *blue curves* correspond to the respective fits). **b** Method B: OpenMalaria simulations of the relationship between prevalence and EIR (model variant R0133 only, other models shown in Additional file 2: Figure S3) for discrete levels of coverage of effective treatment (points) and best fitted model to these data as Hill functions (*curve* for different levels of effective treatment) (Eq. 3). Colour indicates the level of effective treatment (*E*_14_), with *red* 0.001 %, *yellow* 5 %, *light blue* 20 %, *dark blue* 40 %

A previously published statistical model transforming *Pf*PR_2–10_ to EIR [7] (Additional file 7 of that paper), based on an earlier empirical analysis of the relationship of measured EIR values with *Pf*PR_2–10_ [6]:

1 $$x \sim \log {\text{Normal}}\left( {\mu ,\sigma^{2} } \right)$$

where *x* is EIR, *μ* = 1.768 + 7.247*p*, σ = 1.281, and *p*, is *Pf*PR_2–10_. This relationship is independent of the level of access to effective treatment, *E*_14_, and thus does not allow for the effects of case management on the prevalence-EIR relationship. This model allows for statistical uncertainty in both variables *x* and *p* (data and fitted curve shown in Fig. 2a). Scale factors can be used to obtain the EIR estimate that would be obtained with different measurement approaches (e.g. pyrethroid-spraying catches, human landing catches, or both). This method is similar to the method used in previous analyses of the global burden of clinical malaria [11].

#### Method B: dynamic model relating EIR, prevalence, and coverage of treatment

**Fig. 3:**
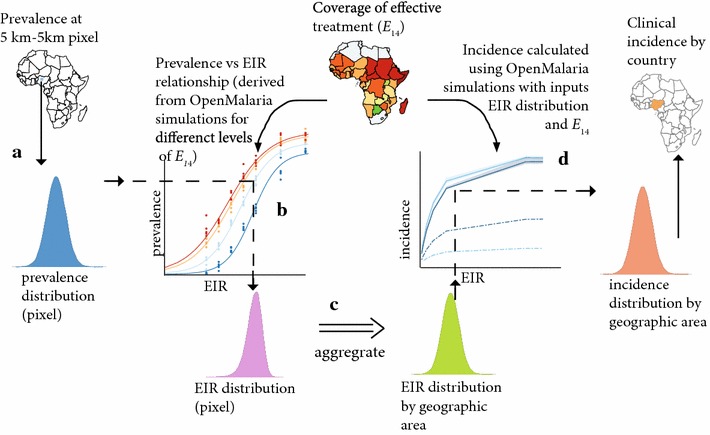
Schematic diagrams of the processes in estimating EIR distributions and disease burden from MAP prevalence. The figure illustrates the steps involved in estimating geographic specific EIR (Method B) and incidence levels of malaria (Methods A and B), which includes the dynamic effect of treatment on transmission, and a dynamic model of clinical incidence. **a**, **b** Illustrate the process of extracting prevalence distributions from MAP [7] by pixel (5 km by 5 km) and converting to distributions of EIR using a statistical relationship relating prevalence and EIR for given levels of effective treatment derived from OpenMalaria simulations. Method A, not illustrated, is simpler in that it does not consider the effect of treatment on transmission, and uses the WMR method for estimating EIR. **c** Illustrates aggregation of these EIR distributions from pixel level to a larger spatial area, such as country level. **d** Illustrates the process of estimating country level burden for a distribution of EIR (derived from either method A or B), namely the EIR distributions are inputs to micro-simulation OpenMalaria with outputs of incidence of clinical cases (and mortality) which are calculated for a given coverage of effective treatment E_14_. The Gaussian distributions in **a**–**d** are illustrative only

Method B uses relationships between EIR and prevalence derived from multiple transmission models of malaria epidemiology and control, incorporating the effects of treatment on the infectious reservoir. The process of translating prevalence to EIR is illustrated in Fig. 3a, b, in essence extracting prevalence distributions at each 5 by 5 km grid from MAP (detailed above, Fig. 3a) and converting to EIR by the fitted relationship from OpenMalaria for a given coverage of effective treatment (Fig. 3b).

The transmission models are six model variants from the OpenMalaria stochastic individual-based model of the dynamics of *P. falciparum* malaria in humans [24] (Table 3), comprising a subset a previously published model ensemble [24] with each model variant including the same sub-model for pathogenesis [23] and case-management [27], but differing by assumptions concerning immunity decay or heterogeneity in transmission or co-morbidity (Table 3). The same parameterizations as used previously [24] were used to capture human demography and the seasonality of transmission. Each model variant has been parameterized by fitting to observed relationships between seasonal patterns of EIR and a range of outcomes, including parasite prevalence [19] and morbidity rates [23] in specific field sites.

**Table 3:** Model-specific parameters for each model variant for the statistical models fits relating OpenMalaria EIR and prevalence among 2–10 year olds

Model	Description of model variant	*K*_1_	*K*_2_	*n*_1_	*n*_2_	*n*_3_	*p*_1_	*p*_2_
R0068	Heterogeneity in transmission: within-host variability	3.40	2.39	−2.44	1.65	1.09	0.90	−0.18
R0131	Immunity decay in effective cumulative exposure	3.68	3.44	0.95	0.68	0.81	0.84	−0.33
R0132	Immunity decay in immune proxies	3.35	3.69	−1.27	2.11	0.72	0.85	−0.45
R0133	Immunity decay in both immune proxies and effective cumulative exposure	3.33	3.49	0.19	1.22	0.86	0.81	−0.39
R0134	Base model (no immunity decay)	4.02	2.67	−1.87	1.52	0.79	0.84	−0.24
R0670	Heterogeneity in susceptibility to co-morbidity	3.51	3.78	1.55	0.43	0.81	0.86	−0.32

A statistical relationship was fit between simulated *Pf*PR_2–10_, *p*, and EIR, *x*, for a given level of effective treatment, *E*_14_, for each model in the ensemble (Fig. 2b illustrates an example of this relationship). These simulated predictions cover a wider range of EIR and prevalence used to parameterize the transmission models originally [17, 24]. The OpenMalaria simulations use a 5-day time step and effective treatment at each 5-day time step, *E*_5_, was obtained from the 14 day estimates using a mapping based on the pattern of fevers over time in malaria-therapy data [28] (sample values shown in Additional file 1: Table S1). A Hill function was fitted by least-squares to the simulation data in order to relate *Pf*PR_2–10_ and EIR, namely:

2 $$p\left( {x,E_{14} } \right) = \frac{{p_{max} x^{{n\left( {E_{14} } \right)}} }}{{K^{{n\left( {E_{14} } \right)}} + x^{{n\left( {E_{14} } \right)}} }},$$

where, *p*_*max*_, *K*, and *n* are functions of *E*_14_. The inverse of relationship Eq. (2), relating EIR to *Pf*PR_2–10_ is given by

3 $$x = K\left( {E_{14} } \right) exp\left[ {\frac{1}{{n\left( {E_{14} } \right)}}ln\left( {\frac{{p\left( {x,E_{14} } \right)}}{{p_ {max}\left( {E_{14} } \right) - p\left( {x,E_{14} } \right)}}} \right)} \right].$$

The functional forms for *x*, *p*_*max*_ and *K* were chosen among exponential, linear, and quadratic options to give the best fit of *p*(*x*, *E*_14_) to the simulated prevalence for different levels of coverage of effective treatment. The selected functions are:

4 $$K\left( {E_{14} } \right) = K_{1} { exp }\left( {K_{2} E_{14} } \right)$$

5 $$n\left( {E_{14} } \right) = n_{1} E_{14}^{2} + n_{2} E_{14} + n_{3}$$

and

6 $$p_{max} \left( {E_{14} } \right) = p_{1} exp\left( {p_{2} E_{14} } \right) .$$

where *K*_1_, *K*_2_, *n*_1_,*n*_2_, *n*_3_, *p*_1_, and *p*_2_ are fitted parameters. Separate parameter sets were fitted for each of the six model variants in the ensemble (values provided in Table 3).

### Estimation of EIR distributions at national level

The prevalence-EIR relationships from method A and B were used to estimate a distribution of EIR for each country from the prevalence surfaces estimated by the MAP for 2010 [7]. Prevalence from the MCMC chains are weighted by each pixel-level value of population, and the percentiles of the distributions obtained by summarizing the whole set of MCMC chains. Corresponding to the *Pf*PR_2–10_ value for pixel j, from MAP, and MCMC iteration i, an EIR estimate, **x**_**j**_^(**i**)^, is obtained. For method B this is

7 $$\mathbf{ x_{j}^{(i)}} = K\left( {E_{14} } \right) exp \left[ {\frac{1}{{n\left( {E_{14} } \right)}}\ln \left( {\frac{{p_{j}^{(i)} }}{{p_{{max} } - p_{j}^{(i)} }}} \right)} \right].$$

The corresponding estimate of the distribution of EIR over the whole country (including non-endemic areas, with EIR = 0) is obtained by binning **x**_**j**_^(**i**)^ into a limited number, *K*, of ranges $X_{ 1} ,X_{ 2} , \ldots ,X_{K}$, and population weighting. Aggregating the estimates from the whole set of *T* sampled values of *p*_*j*_^(*i*)^ from the MCMC chains, each range is assigned probability:

8 $${ \Pr }\left( {X_{k} } \right) = \frac{{\mathop \sum \nolimits_{i} \mathop \sum \nolimits_{j} \left( {N_{j} {\text{I}}\left({ \mathbf{{{x}}_{{j}}^{{\left( {{i}} \right)}}} \in X_{k} } \right)} \right)}}{{T\left( {\mathop \sum \nolimits_{j} N_{j} } \right)}},$$

and I(**x**_**j**_^(**i**)^ ∊ *X*_*k*_) is an indicator taking value 1 if** x**_**j**_^(**i**)^ is in range *X*_*k*_ and zero otherwise. Here *N*_*j*_ is the population assigned to the pixel as determined by the gridded population of the world [29, 30]. For computational convenience we carried out the summation over *i* before summing over *j*.

The resulting distributions describe the proportion of each country’s population that one would expect to be living at a given level of prevalence. In many of the countries analysed, a proportion of the gridded population from [30] falls outside the boundary of the area defined by MAP as being within the spatial limits of endemic malaria transmission [7]. This proportion of the population was assigned an EIR of zero.

Two different estimates of the transmission distribution per geographic area were calculated by estimation method B, to examine sensitivity to the estimated level of access to effective treatment. To capture the situation before recent scale-up of artemisinin combination treatment (ACT), a common value of access to effective treatment for all countries was used assumed and at a value previously used in OpenMalaria simulations [31]. This value equates to approximately 15 % of all malaria cases receiving treatment resulting in parasitological cure. In addition, analyses were conducted using country-specific estimates of access to effective treatment [25]. Country levels of coverage of effective treatment are listed Table 2 and illustrated by map in Additional file 2: Figure S1 and in Fig. 3b.

### Burden of disease

National level estimates of the incidence of clinical malaria were projected from the EIR distributions derived from Method A and Method B using OpenMalaria simulations. These incorporate dynamic models of clinical incidence and treatment parameterized with Senegalese and Tanzanian data [23, 31] and models for severe disease and mortality [22], and hence provide clinical incidence estimates as an extension of EIR estimation (process illustrated in Fig. 3d). Separate estimates were made using the EIR estimates with Method A, those from with Method B with E_14_ = 0.15, and those made with Method B with country specific E_14_ values.

Estimated burden, via clinical incidence, derived by both methods was compared with those national level estimates of clinical malaria from the WMR. For most sub-Saharan African countries, these use a standard empirical relationship between clinical incidence and endemicity. Clinical incidence values were assigned to each endemicity level based on estimates of the numbers of events recorded in longitudinal surveys of febrile malaria episodes in children, detected either actively or passively [32–34], established independently of effects of treatment rates [12]. For countries with low endemicity, WMR uses national surveillance data to estimate burden, with adjustments to allow for incomplete reporting.

## Results

### National level prevalence distributions

National levels of *Pf*PR_2–10_ aggregated at country level after extracting from MAP [7] posterior distributions at each 5 by 5 km pixel illustrate high average levels of *Pf*PR_2–10_ in 2010 in many African countries but for many countries also the wide distribution of prevalence levels (values summarized in Table 4 and Additional file 1: Table S3 and shown as distributions in Additional file 2: Figure S2). Regional differences, local variation, and uncertainty within areas all contribute differently to the overall distributions, with the average levels of transmission highest in West and Central Africa. Much of Namibia, Botswana, and South Africa, and also several Sahelian countries are malaria free, as are highland areas of East Africa. Some of the variation is also a result of differences in the extent of recent intervention programmes. In some countries, intervention programmes have had little impact on 2010 prevalence (e.g. Benin, Burkina Faso, Côte d’Ivoire), while elsewhere prevalence has been considerably reduced in the last decade (e.g. Senegal, Tanzania, Zambia), or much of the population lives in areas on the margins of stable transmission (Somalia, North Sudan). The location of some major urban centres such as Nairobi and Lusaka at relatively high altitudes, with low transmission, strongly influences some of these profiles.

**Table 4:** Prevalence distributions, summarized for each country: estimated prevalence (mean, median and quartiles) for 43 sub-Saharan Africa estimated from MAP prevalence posteriors at 5 km by 5 km grids, aggregated to country level and weighted by population. Mean EIR estimates for Method A aand Method B assuming effective coverage (*E*_14_) of 15 % for all countries

Country	Country code	Mean prevalence	Prevalence median	Weighted mean EIR for Method A	Weighted median EIR for Method B with *E*_14 _of 15 %
Angola	ago	0.284	0.160	18.09	1.37
Benin	ben	0.467	0.440	35.55	7.55
Botswana	bwa	0.018	0.000	0.39	0.00
Burkina Faso	bfa	0.622	0.680	61.15	41.71
Burundi	bdi	0.114	0.001	3.57	0.25
Cameroon	cmr	0.457	0.440	34.07	7.55
Central African Republic	caf	0.462	0.440	35.73	7.55
Chad	tcd	0.265	0.120	14.67	1.37
Comoros	com	0.302	0.160	19.71	1.65
Congo	cog	0.318	0.240	18.07	2.42
Democratic Republic of Congo	cod	0.344	0.240	23.32	2.42
Cote d’Ivoire	civ	0.514	0.520	40.56	11.04
Djibouti	dji	0.001	0.000	0.03	0.00
Equatorial Guinea	gnq	0.520	0.520	41.78	13.35
Eritrea	eri	0.018	0.001	0.49	0.04
Ethiopia	eth	0.023	0.000	0.57	0.03
Gabon	gab	0.432	0.400	29.59	6.25
The Gambia	gmb	0.092	0.001	1.52	0.36
Ghana	gha	0.337	0.240	21.40	2.42
Guinea	gin	0.322	0.200	19.06	2.42
Guinea Bissau	gnb	0.105	0.040	2.18	0.36
Kenya	ken	0.068	0.001	2.80	0.04
Liberia	lbr	0.395	0.360	19.65	4.27
Madagascar	mdg	0.308	0.160	22.87	1.65
Malawi	mwi	0.355	0.280	22.02	2.92
Mali	mli	0.483	0.480	38.53	9.13
Mauritania	mrt	0.058	0.000	2.58	0.04
Mozambique	moz	0.391	0.320	30.07	3.53
Namibia	nam	0.095	0.001	3.66	0.05
Niger	ner	0.285	0.160	15.30	1.65
Nigeria	nga	0.429	0.400	30.51	6.25
Rwanda	rwa	0.023	0.000	0.48	0.04
Sao Tome Principe	stp	0.122	0.040	2.22	0.44
Senegal	sen	0.076	0.001	1.86	0.20
Sierra Leone	sle	0.401	0.360	25.55	4.27
Somalia	som	0.039	0.001	0.77	0.08
North Sudan	sdn	0.072	0.001	3.65	0.07
South Sudan	ssd	0.168	0.001	10.24	0.25
Tanzania	tza	0.172	0.040	8.16	0.44
Togo	tgo	0.440	0.400	32.62	6.25
Uganda	uga	0.399	0.360	25.97	4.27
Zambia	zmb	0.165	0.040	7.18	0.44
Zimbabwe	zwe	0.044	0.001	1.12	0.07

### Modelled relationships between EIR and prevalence

Where *Pf*PR_2–10_ is high, the OpenMalaria models predict on average a slightly higher EIR at a given prevalence than does the empirical model (method A), with relatively little influence of effective treatment (*E*_14_) (Fig. 2a, b). The fitted prevalence to EIR relationships for Method B are shown in Fig. 2b (model variant R133) and Additional file 2: Figure S3 (all 6 model variants). The six model variants all predict broadly similar, but nevertheless distinct, prevalence-EIR relationships. The general pattern for Method A is for prevalence to increase steeply with EIR at low transmission levels, but to saturate at higher transmission (Fig. 2a). The considerable variation around the best fitting curve for Method A, after adjusting for the different EIR measurement techniques used, is treated as random variation that contributes to uncertainty in the estimate of EIR from prevalence. This analysis does not allow for variations in the coverage or effectiveness of case management in the different studies, however such variation could account for much of this unexplained dispersion (compare with Fig. 2b). At lower transmission levels the fitted curves for Method B (OpenMalaria) vary considerably with *E*_14_, suggesting that effectiveness of case management is a particularly important driver of prevalence in such settings, with Method B estimating lower EIR at a given prevalence than the empirical model unless *E*_14_ is high (Fig. 2b). This is partly because Method B constrains estimated EIR to be zero at zero prevalence, while the empirically-based Method A does not capture or force this constraint.

### National level EIR distributions

**Fig. 4:**
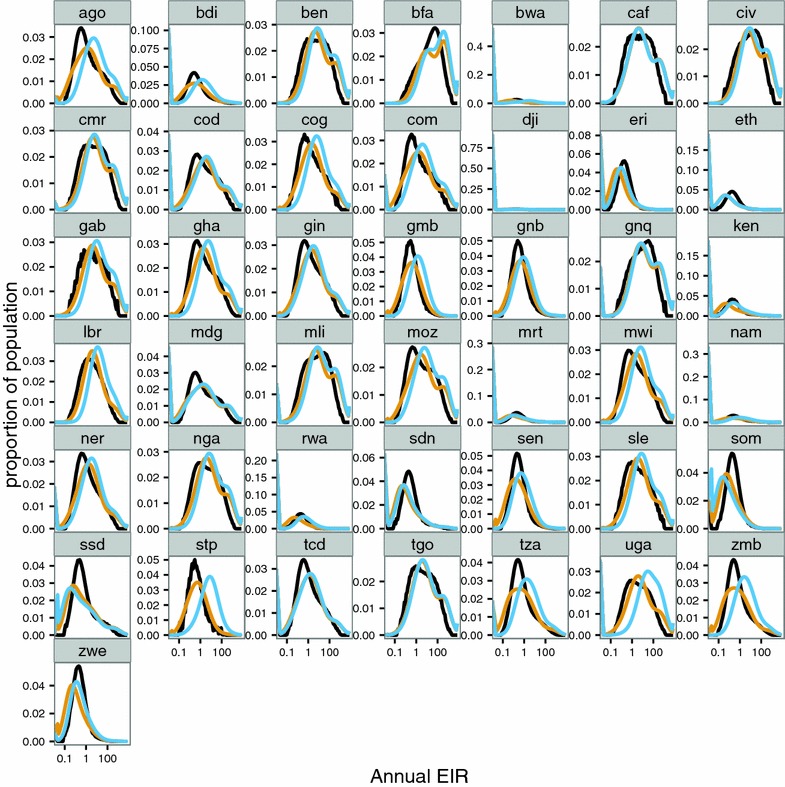
Distribution of EIR for each of the 43 countries. Distribution of EIR (including non-endemic areas which are assigned values of 0) for each of the 43 countries. Calculated from MAP using both the empirical model (Method A) (*black*); the simulation models (Method B) with a common value for access to care (*yellow*) ($$E_{14} = 0.15)$$; and country-specific values of $$E_{14}$$(*blue*). Countries are indicated by country code

**Fig. 5:**
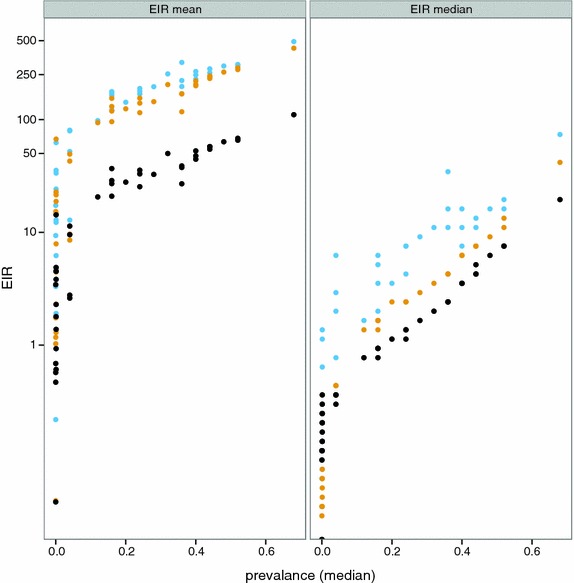
Relationship of estimated average EIR to prevalence at country level. Calculated from MAP using both the empirical model (Method A) (*black*); the simulation models (Method B) with a common value for access to care, (*yellow*) (E_14_ = 0.15); and country-specific values of ${\text{E}}_{14}$(*blue*)

The differences between the two relationships for prevalence and EIR are reflected in the estimated distributions of EIR by county (Fig. 4; Additional file 2: Figure S4). The EIR distributions are generally much more highly skewed than are the prevalence distributions. The distributions obtained with the empirical model (Method A: Fig. 4; Table 4 and Additional file 1: Table S4) and with the simulation models (Method B) that assume $E_{14} = 0.15$ (Table 4 and Additional file 1: Table S5) are similar to each other for most countries, though the estimated median EIRs are generally somewhat higher for the estimates from the simulation models (Fig. 5). Where effectiveness of case management is high, the country specific assumptions for system effectiveness make substantial differences to the estimated EIR distributions (Fig. 4; Table 2; Additional file 2: Figure S2). In these countries, notably Zambia, Tanzania, São Tomé and Principe, the EIR distribution shifts to the right when the country-specific value of ${\text{E}}_{14}$ is used, reflecting lower prevalence than in a situation with the same EIR pattern, but less effective case management. The estimate of median EIR for these countries is thus much higher when country-specific effectiveness is considered. Conversely, in a few countries, where median prevalence is low, and case management is also poor, the estimated EIR distribution allowing for country-specific effectiveness is shifted slightly to the left (e.g. both South and North Sudan).

The EIR distributions are highly skewed, so that the arithmetic means are much higher than the medians (Figs. 4, 5). Except in some cases where prevalence is very low, the average EIR is higher when there is allowance for treatment (Method B), with a much larger shift in the mean than in the median of the distribution. When country specific *E*_14_ values are used (which are mostly higher than the 15 % shown in yellow), this makes little difference to the mean EIR, but substantial differences to the medians, reflecting the stronger relationship between treatment rates and prevalence when EIR is low, than when EIR is high.

### National levels of burden of disease

**Fig. 6:**
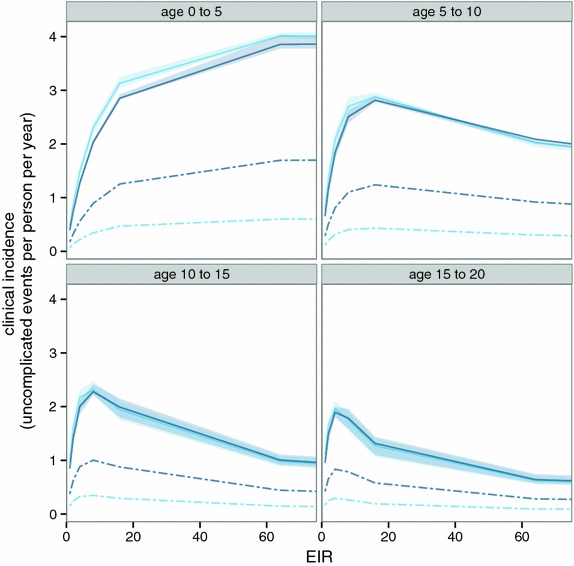
Models of the relationship between EIR and clinical incidence. Incidence of clinical episodes by EIR in OpenMalaria models with *light blue* $E_{14}$ = 0.15 and *dark blue* $E_{14}$ = 0.45. The *continuous lines* indicate the mean prediction of the overall incidence. The *shading around the continuous lines* indicates the range of predictions made from simulations with different model variants and random number seeds. The *dashed lines* indicate the incidence of clinical episodes that are treated [31]

The OpenMalaria simulations predict that steady state clinical incidence (over all ages) increases linearly with EIR in low transmission settings, tending to plateau at high EIR (with a suggestion, driven by the specific Senegalese data used to parameterize the models for older children and adults [35] that there may be a maximum in the curve at high prevalence). The initial slope is greater when $E_{14}$ is higher, but the plateau occurs at a similar level of incidence irrespective of effective treatment level. These patterns are a consequence of the age-specific relationships between incidence and EIR shown in Fig. 6.

**Fig. 7:**
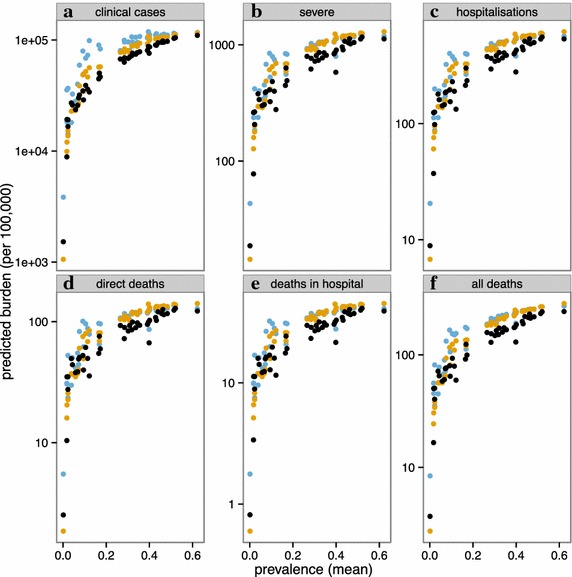
Predicted incidence of clinical events by national level average EIR. Predicted incidence of clinical events estimated from empirical model (Method A) (*black*); the simulation models (Method B) with a common value for access to care (*yellow*) (E_14_ = 0.15); and country-specific values of $E_{14}$(*blue*). **a** uncomplicated clinical episodes (cases); **b** severe malaria episodes; **c** hospitalizations; **d** deaths directly attributable to malaria; **e** hospital deaths; **f** all malaria deaths (including those with co-morbidities). All rates are expressed as events per 100,000 person years at risk over all ages of hosts. The model and parameters for severe disease and mortality follows Ross et al. [22], with a common hospitalization rate assumed for severe disease across all countries

When these models are used to infer country-specific incidence of clinical malaria, there is a clear increase in incidence with average prevalence at the country level (Fig. 7), and no plateau is reached because even the countries with highest average transmission have only small populations in the very high EIR categories (Table 4 and Additional file 1: Table S2). The relationships between country-level EIR and estimated clinical malaria incidence are similar, irrespective of whether the EIR is estimated by Method A or Method B. Similarly, Method B estimates similar relationships between country-level EIR and clinical malaria incidence, irrespective of whether a common value, or a country specific estimate is used for the effectiveness of case management.

**Fig. 8:**
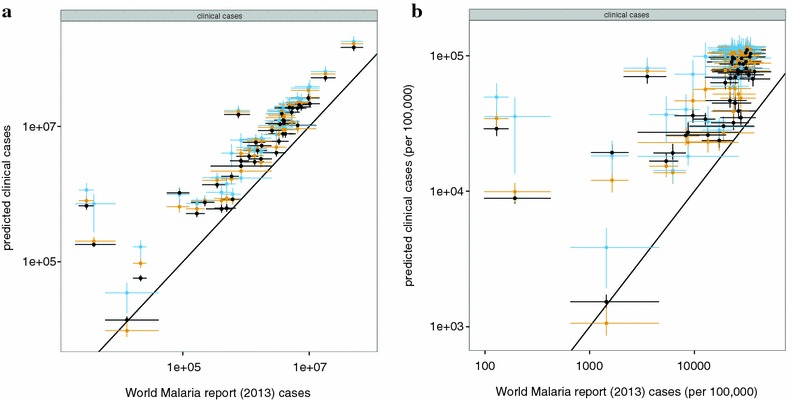
Numbers of episodes per annum estimated using different approaches. Numbers of clinical cases; **b** incidence rate (episodes or cases per 100,000 person-year). *Black points*: based on EIR estimates calculated using Method A; *yellow points*: based on EIR estimates made using Method B with a common value for access to care ($$E_{14} = 0.15)$$; *blue points*: based on EIR estimates made using Method B with country-specific values of $$E_{14}$$(*blue*). The *diagonal line* corresponds to a 1:1 relationship; the *horizontal and vertical lines* represent minimum and maximum ranges

Country-specific estimates of clinical incidence using country EIR distributions is compared to published malaria cases from the World Malaria Report [14] (Fig. 8; Additional file 2: Figure S5). In general, projections of incidence using EIR derived from method B produces higher predictions than using EIR from method A, but in both cases the simulation models predict substantially more episodes of malaria than the cases reported in the World Malaria Report 2013 (Fig. 8a). There is also a much less steep relationship between the incidence rate and the overall burden (Fig. 8b). This can be explained by the empirical relationships between prevalence and case incidence used by WMR [12], which refer back to field research carried out prior to the widespread use of ACT [33, 36], and therefore do not allow for level of treatment. Moreover, only clinical episodes in children under 5 years of age are considered. The effect of high levels of treatment on reducing prevalence leads to much higher ratios of case-incidence to prevalence ratio than it would be without treatment (Fig. 7). OpenMalaria correctly predicts that in low transmission countries the majority of the clinical burden is in older age groups (Fig. 6). The difference between the methods is particularly evident for low-burden countries Namibia and Botswana, for which very low case numbers are reported by the WMR, with estimates based on adjustments to surveillance data.

## Discussion

It has been incontrovertible since Laveran’s first studies of the malaria parasite [37] that effective treatment of clinical malaria results in clearance of blood-stage parasites. Treatment lowers the overall prevalence associated with malaria (or other parasites [38]), the infectiousness of the human population and the transmission level [31], both of which synergize with effects of other interventions on transmission. Most immediately, effective treatment reduces the length of illness and the incidence adverse outcomes, including severe disease, neurological sequelae, and death. This reduces the burden of disease that can potentially be averted by other interventions. All these effects need to be considered in estimating current burden of disease, in analyses of the impact of case management and treatment on the burden of disease, and analyses of treatment modifies the public health impact achievable with other curative and preventive interventions. Results presented here clearly indicate that incorporating the dynamic effects of treatment is essential for valid estimation of EIR, of clinical incidence itself, and of downstream outcomes including the incidence of severe disease and mortality rates, with substantively differences in estimates when included or excluded.

Overall, the model-based method proposed in this work (method B) provides estimates of transmission intensity, as measured by EIR, that are somewhat higher than those estimated by method A [7], especially in low endemicity countries and where case management is relatively effective. The downstream country level clinical incidence estimates are also higher than those previously reported in the World Malaria Report [13]. At the country level, allowing for uncertainty in the inputs makes a substantial difference to average values of both EIRs and disease rates, as a result of the skewness of their distributions. This means that incorporating uncertainty and spatial variation into the estimation has important consequences for both burden estimates and prediction of average health impacts of interventions, which in general vary non-linearly with EIR. The OpenMalaria models also predict, as one would expect, that the effectiveness of uncomplicated malaria treatment has substantial impact on the incidence of severe disease and malaria mortality.

Preventive interventions like insecticide treated nets (ITNs), which affect prevalence only via their impact on exposure, do not change the relationship between exposure and prevalence. Consequently, coverages of preventive interventions can be useful covariates for estimating EIR or prevalence surfaces where direct measurements are sparse, but the coverage of these interventions are not directly relevant when making estimations of disease burden from prevalence. In contrast, treatment of malaria reduces the prevalence at a given level of EIR, by preventing infections from persisting, thus modifying the relationship between the two metrics (Fig. 1). So the same prevalence can result from very different average exposures depending on the level of treatment, and the effective coverage of case management (like the degree of transmission heterogeneity [2]) should be taken account in modeling the relationships between EIR and prevalence.

Nevertheless, at least in high endemicity settings, prevalence remains the best measure on which to base geographically specific models of malaria transmission. This is because prevalence data are actively collected based on representative sampling of populations, are widely available, have been compiled into publicly accessible databases [7, 39], and have been analysed using geostatistical models to produce high resolution maps of the distribution of infection in space [7, 40]. In most sub-Saharan African countries prevalence is therefore likely to remain the main metric used in deciding when and where to distribute or target interventions. In low transmission settings such as those in Asia, Latin America, and selected African countries the annual parasite index (API) rather than the prevalence is the main metric used for monitoring and evaluation, and WMR has estimated burden in these countries using an API-based algorithm [12]. Prevalence-EIR-treatment relationships in such low transmission settings can be captured by relatively simple empirical mathematical models [4]. However in areas of moderate or high transmission it is important to allow for effects of superinfection and natural immunity, and thus mechanistic models that account for dynamics of immunity are needed.

The use of simulation models that take both prevalence and treatment rates as inputs provides a generalizable way of generating national level estimates of transmission and disease burden, applicable across the range of transmission intensities. This generalizability will be important for monitoring progress as malaria is further controlled to the point where measurement of API becomes the main metric used by many more country programmes. The approach will capture in a natural way the transitions between the different metrics, and the age shifts in the pattern of disease where transmission rates change [41, 42]. The approach can be made more robust by employing a larger ensemble including other simulation models with different assumptions about transmission heterogeneity, immunity, and pathogenesis [10, 43]. For the method to provide the best estimates of malaria attributable mortality, geographical variation in access to appropriate in-patient treatment of severe disease also needs to be taken into account.

Previous methodologies for estimating burden have applied both estimates of intervention protective efficacy derived from meta-analyses of controlled trials and/or household survey data, leading to circular reasoning. Local variability has also been ignored [1], in particular variations in access, compliance, or adherence, and also the medium- and long-term dynamics resulting from intervention-induced reductions in transmission, which include shifts of disease into older age groups [41, 42]. The burden estimation procedures proposed in this paper will allow empirical analysis of the relationships between intervention coverage and burden independently of field trial results and conditional on all these factors. This will provide a basis for assessing the impacts of both preventive and curative interventions on an equivalent basis, ensuring correct attribution of the effects of different interventions. The method can be extended to give time-dependent estimates of burden by using time-period specific input data. By linking these to intervention coverage, this will provide valid estimates of intervention impacts in time and space. Although results are presented only at country level in this work, this methodology can, in principle, be applied to any level of spatial aggregation. However, applying the approach to data disaggregated in smaller spatial units would raise additional methodological issues, as the simulation models are parameterized mainly using village-level data.

This paper demonstrates the dual importance of capturing the effects of treatment when estimating disease burden based on infection prevalence: to both improve the accuracy of those estimates and to correctly quantify the impact of treatment on reduced malaria transmission and illness. These insights are currently being incorporated into a revised WHO methodology that will lead to more refined burden estimates and ultimately better information for national and international malaria control decision-making processes.

## Additional files

10.1186/s12936-015-0864-3 This file includes additional Tables that support and expand some of the results in the main text, but whose inclusion would detract from the main argument.
10.1186/s12936-015-0864-3 This file includes additional Figures of results that support and expand some of the results in the main text, but whose inclusion would detract from the main argument.
