# Supplementary material for: Distribution of malaria exposure in endemic countries in Africa considering country levels of effective treatment
Source: Malar J. 2015 Oct 5;14:384. doi: 10.1186/s12936-015-0864-3 (PMC4595196; doi:10.1186/s12936-015-0864-3)
Supplement: Supplementary file 1 — 10.1186/s12936-015-0864-3 This file includes additional Tables that support and expand some of the results in the main text, but whose inclusion would detract from the main argument. [file 12936_2015_864_MOESM1_ESM.pdf]

Additional File 1 – Tables

Distributions of malaria exposure in endemic countries in Africa considering country levels of effective treatment

Melissa A. Penny*, Nicolas Maire, Caitlin Bever, Peter Pemberton-Ross, Olivier J.T. Briët, David L. Smith, Peter W. Gething, Thomas A. Smith

* Correspondence: melissa.penny@unibas.ch

This Additional file includes additional results that support and expand some of the results in the main text, but whose inclusion would detract from the main argument

Additional File 1: Table S1: Mapping of 14 day effective coverage onto 5 day probability of effective treatment (required input to OpenMalaria)

**Effective coverage E_{14} (%)**	**OpenMalaria 5 day probability $E_{5\,(\%)}$**
0.1	0.04
1	0.37
5	1.82
10	3.56
15	5.16
20	7.25
25	9.72
30	12.27
35	14.88
40	17.82
45	20.93
50	24.12
55	27.15
60	30.30
65	35.67
70	41.65
75	46.46
80	53.19

Additional File 1: Table S2. EIR distributions obtained with the simulation models (Method B) with country-specific effectiveness of case management(E_{14})

country	country code	Weighted mean EIR	E_{14} (%) [1]	EIR midpoint (range)								
				0 (0-0.071)	0.1 (0.071-0.71)	1 (0.71-1.41)	2 (1.41-2.83)	4 (2.83-5.66)	8 (5.66-11.3)	16 (11.3-45.3)	64 (45.3-181)	256 (181-∞)
Angola	ago	49.8	49.5	0.003	0.165	0.094	0.082	0.117	0.115	0.164	0.110	0.150
Benin	ben	72.8	34.5	0.000	0.094	0.072	0.071	0.109	0.114	0.173	0.133	0.234
Botswana	bwa	5.3	72.0	0.529	0.144	0.057	0.051	0.069	0.059	0.061	0.022	0.008
Burkina Faso	bfa	118.1	35.9	0.000	0.031	0.035	0.040	0.071	0.086	0.161	0.174	0.404
Burundi	bdi	13.5	42.4	0.103	0.290	0.127	0.096	0.117	0.094	0.101	0.042	0.030
Cameroon	cmr	67.4	29.9	0.003	0.117	0.078	0.074	0.111	0.113	0.165	0.124	0.216
Central African Republic	caf	61.5	18.9	0.000	0.185	0.088	0.075	0.105	0.100	0.140	0.107	0.199
Chad	tcd	27.8	20.7	0.030	0.344	0.110	0.083	0.103	0.087	0.103	0.060	0.082
Comoros	com	46.5	39.2	0.015	0.202	0.097	0.082	0.113	0.107	0.145	0.096	0.143
Congo	cog	49.0	44.1	0.000	0.130	0.089	0.084	0.126	0.128	0.183	0.116	0.144
Democratic Republic of Congo	cod	47.4	30.2	0.039	0.204	0.092	0.078	0.108	0.102	0.138	0.093	0.148
Cote d'Ivoire	civ	78.8	29.2	0.000	0.083	0.066	0.066	0.104	0.111	0.173	0.140	0.256
Djibouti	dji	0.2	48.6	0.915	0.051	0.014	0.008	0.007	0.003	0.002	0.001	0.000
Equatorial Guinea	gnq	76.8	24.0	0.018	0.092	0.068	0.066	0.102	0.106	0.163	0.133	0.251
Eritrea	eri	1.1	25.2	0.081	0.785	0.058	0.027	0.022	0.013	0.010	0.003	0.001
Ethiopia	eth	1.0	16.4	0.189	0.708	0.040	0.020	0.019	0.011	0.009	0.003	0.001
Gabon	gab	71.7	40.8	0.000	0.073	0.064	0.067	0.110	0.122	0.194	0.146	0.225
The Gambia	gmb	7.3	40.8	0.000	0.340	0.160	0.120	0.142	0.106	0.096	0.028	0.008
Ghana	gha	52.3	43.0	0.000	0.138	0.091	0.084	0.123	0.122	0.171	0.112	0.159
Guinea	gin	39.7	28.4	0.000	0.243	0.106	0.087	0.118	0.107	0.137	0.084	0.119
Guinea Bissau	gnb	6.3	29.5	0.000	0.445	0.156	0.107	0.116	0.080	0.068	0.020	0.009
Kenya	ken	7.7	37.8	0.187	0.489	0.092	0.053	0.054	0.041	0.045	0.021	0.019
Liberia	lbr	60.2	47.4	0.000	0.044	0.056	0.066	0.120	0.144	0.238	0.160	0.173
Madagascar	mdg	42.0	22.4	0.046	0.316	0.090	0.069	0.089	0.079	0.103	0.074	0.134
Malawi	mwi	54.5	37.7	0.000	0.128	0.085	0.080	0.121	0.123	0.178	0.119	0.166
Mali	mli	76.0	29.2	0.014	0.098	0.068	0.066	0.102	0.107	0.165	0.133	0.247
Mauritania	mrt	5.4	24.5	0.286	0.496	0.064	0.037	0.038	0.027	0.026	0.012	0.014
Mozambique	moz	65.8	38.7	0.000	0.144	0.081	0.073	0.107	0.107	0.158	0.120	0.211
Namibia	nam	11.3	39.6	0.331	0.264	0.087	0.061	0.073	0.059	0.066	0.031	0.028
Niger	ner	35.3	33.1	0.019	0.215	0.108	0.092	0.125	0.114	0.143	0.082	0.102
Nigeria	nga	65.7	40.9	0.000	0.111	0.077	0.074	0.113	0.117	0.173	0.127	0.208
Rwanda	rwa	2.2	41.5	0.221	0.465	0.117	0.066	0.060	0.036	0.027	0.007	0.002
Sao Tome Principe	stp	25.8	68.8	0.000	0.135	0.090	0.090	0.145	0.155	0.223	0.112	0.051
Senegal	sen	5.8	34.0	0.000	0.515	0.144	0.093	0.098	0.066	0.057	0.018	0.010
Sierra Leone	sle	61.0	39.3	0.000	0.102	0.076	0.075	0.118	0.125	0.188	0.130	0.188
Somalia	som	1.1	7.8	0.021	0.849	0.049	0.027	0.024	0.015	0.011	0.003	0.001
North Sudan	sdn	7.0	20.7	0.063	0.711	0.063	0.036	0.037	0.027	0.028	0.015	0.020
South Sudan	ssd	17.0	9.2	0.000	0.652	0.068	0.046	0.054	0.044	0.052	0.033	0.052
Tanzania	tza	25.1	46.9	0.019	0.290	0.121	0.093	0.117	0.102	0.125	0.066	0.068
Togo	tgo	58.9	19.8	0.000	0.170	0.091	0.080	0.112	0.106	0.146	0.106	0.188
Uganda	uga	89.7	67.1	0.036	0.035	0.033	0.038	0.074	0.099	0.206	0.195	0.284
Zambia	zmb	26.9	52.7	0.000	0.238	0.123	0.100	0.131	0.117	0.146	0.077	0.070
Zimbabwe	zwe	2.8	27.0	0.006	0.741	0.090	0.049	0.046	0.030	0.026	0.008	0.005

Additional File 1: Table S3. Prevalence distributions, summarized for each country: Estimated prevalence (mean, median and quartiles) for 43 sub-Saharan Africa estimated from MAP prevalence posteriors at 5km by 5 km grids, aggregated to country level and weighted by population.

country	country code	mean prevalence	prevalence median	prevalence 25% quartile	prevalence 75% quartile
Angola	ago	0.284	0.160	0.001	0.480
Benin	ben	0.467	0.440	0.160	0.720
Botswana	bwa	0.018	0.000	0.000	0.001
Burkina Faso	bfa	0.622	0.680	0.400	0.840
Burundi	bdi	0.114	0.001	0.000	0.120
Cameroon	cmr	0.457	0.440	0.160	0.720
Central African Republic	caf	0.462	0.440	0.160	0.720
Chad	tcd	0.265	0.120	0.001	0.440
Comoros	com	0.302	0.160	0.001	0.520
Congo	cog	0.318	0.240	0.040	0.520
Democratic Republic of Congo	cod	0.344	0.240	0.040	0.600
Cote d'Ivoire	civ	0.514	0.520	0.240	0.760
Djibouti	dji	0.001	0.000	0.000	0.000
Equatorial Guinea	gnq	0.520	0.520	0.240	0.760
Eritrea	eri	0.018	0.001	0.000	0.001
Ethiopia	eth	0.023	0.000	0.000	0.001
Gabon	gab	0.432	0.400	0.160	0.680
The Gambia	gmb	0.092	0.001	0.001	0.120
Ghana	gha	0.337	0.240	0.040	0.560
Guinea	gin	0.322	0.200	0.040	0.520
Guinea Bissau	gnb	0.105	0.040	0.001	0.120
Kenya	ken	0.068	0.001	0.000	0.001
Liberia	lbr	0.395	0.360	0.160	0.560
Madagascar	mdg	0.308	0.160	0.001	0.560
Malawi	mwi	0.355	0.280	0.080	0.600
Mali	mli	0.483	0.480	0.160	0.760
Mauritania	mrt	0.058	0.000	0.000	0.001
Mozambique	moz	0.391	0.320	0.040	0.680
Namibia	nam	0.095	0.001	0.000	0.080
Niger	ner	0.285	0.160	0.001	0.480
Nigeria	nga	0.429	0.400	0.120	0.680
Rwanda	rwa	0.023	0.000	0.000	0.001
Sao Tome Principe	stp	0.122	0.040	0.001	0.160
Senegal	sen	0.076	0.001	0.000	0.080
Sierra Leone	sle	0.401	0.360	0.120	0.640
Somalia	som	0.039	0.001	0.000	0.001
North Sudan	sdn	0.072	0.001	0.000	0.001
South Sudan	ssd	0.168	0.001	0.000	0.240
Tanzania	tza	0.172	0.040	0.001	0.240
Togo	tgo	0.440	0.400	0.120	0.680
Uganda	uga	0.399	0.360	0.080	0.640
Zambia	zmb	0.165	0.040	0.001	0.240
Zimbabwe	zwe	0.044	0.001	0.000	0.001

Additional File 1: Table S4: EIR distributions obtained with the empirical model (Method A)

country	country code	weighted mean EIR	weighted median EIR	EIR 25% quartile	EIR 75% quartile.
Angola	ago	18.09	0.77	0.20	7.55
Benin	ben	35.55	5.16	0.64	34.50
Botswana	bwa	0.39	0.00	0.00	0.17
Burkina Faso	bfa	61.15	19.52	2.92	89.16
Burundi	bdi	3.57	0.25	0.07	0.94
Cameroon	cmr	34.07	4.27	0.64	34.50
Central African Republic	caf	35.73	4.27	0.64	34.50
Chad	tcd	14.67	0.77	0.17	5.16
Comoros	com	19.71	0.94	0.20	9.13
Congo	cog	18.07	1.13	0.25	9.13
Democratic Republic of Congo	cod	23.32	1.37	0.25	13.35
Cote d'Ivoire	civ	40.56	7.55	1.13	41.71
Djibouti	dji	0.03	0.00	0.00	0.00
Equatorial Guinea	gnq	41.78	7.55	1.13	50.44
Eritrea	eri	0.49	0.14	0.05	0.36
Ethiopia	eth	0.57	0.12	0.03	0.36
Gabon	gab	29.59	3.53	0.53	23.60
The Gambia	gmb	1.52	0.30	0.10	0.77
Ghana	gha	21.40	1.37	0.25	11.04
Guinea	gin	19.06	1.13	0.25	9.13
Guinea Bissau	gnb	2.18	0.30	0.12	0.94
Kenya	ken	2.80	0.14	0.03	0.53
Liberia	lbr	19.65	2.42	0.53	13.35
Madagascar	mdg	22.87	0.94	0.17	11.04
Malawi	mwi	22.02	1.65	0.30	13.35
Mali	mli	38.53	6.25	0.77	41.71
Mauritania	mrt	2.58	0.10	0.00	0.44
Mozambique	moz	30.07	2.00	0.30	23.60
Namibia	nam	3.66	0.12	0.00	0.64
Niger	ner	15.30	0.94	0.20	6.25
Nigeria	nga	30.51	3.53	0.53	28.53
Rwanda	rwa	0.48	0.12	0.02	0.36
Sao Tome Principe	stp	2.22	0.36	0.12	1.13
Senegal	sen	1.86	0.25	0.10	0.64
Sierra Leone	sle	25.55	2.42	0.44	19.52
Somalia	som	0.77	0.17	0.07	0.53
North Sudan	sdn	3.65	0.20	0.07	0.53
South Sudan	ssd	10.24	0.36	0.12	1.65
Tanzania	tza	8.16	0.36	0.12	1.65
Togo	tgo	32.62	3.53	0.53	28.53
Uganda	uga	25.97	2.42	0.36	19.52
Zambia	zmb	7.18	0.36	0.12	1.65
Zimbabwe	zwe	1.12	0.20	0.08	0.53

Additional File 1: Table S5: Estimated EIR (mean, median and quartiles) for 43 sub-Saharan Africa estimated via method B assuming effective coverage (E_{14}) of 15% for all countries.

country	country code	weighted mean EIR	weighted median EIR	EIR 25% quartile	EIR 75% quartile.
Angola	ago	32.04	1.37	0.20	11.04
Benin	ben	61.90	7.55	1.37	89.16
Botswana	bwa	0.68	0.00	0.00	0.05
Burkina Faso	bfa	102.90	41.71	4.27	336.87
Burundi	bdi	6.83	0.25	0.04	1.37
Cameroon	cmr	59.73	7.55	1.13	73.74
Central African Republic	caf	62.15	7.55	1.13	89.16
Chad	tcd	26.54	1.37	0.20	9.13
Comoros	com	34.95	1.65	0.20	16.14
Congo	cog	32.43	2.42	0.44	16.14
Democratic Republic of Congo	cod	41.02	2.42	0.36	23.60
Cote d'Ivoire	civ	70.77	11.04	2.00	130.35
Djibouti	dji	0.04	0.00	0.00	0.00
Equatorial Guinea	gnq	73.27	13.35	2.00	130.35
Eritrea	eri	0.74	0.04	0.01	0.14
Ethiopia	eth	0.92	0.03	0.01	0.14
Gabon	gab	52.92	6.25	1.13	50.44
The Gambia	gmb	2.94	0.36	0.08	1.37
Ghana	gha	37.77	2.42	0.44	19.52
Guinea	gin	34.42	2.42	0.36	16.14
Guinea Bissau	gnb	4.11	0.36	0.10	1.65
Kenya	ken	4.92	0.04	0.01	0.30
Liberia	lbr	35.74	4.27	1.13	23.60
Madagascar	mdg	39.63	1.65	0.14	19.52
Malawi	mwi	39.43	2.92	0.53	23.60
Mali	mli	66.88	9.13	1.37	107.80
Mauritania	mrt	4.65	0.04	0.00	0.30
Mozambique	moz	51.99	3.53	0.53	50.44
Namibia	nam	7.00	0.05	0.00	0.77
Niger	ner	27.51	1.65	0.30	11.04
Nigeria	nga	53.76	6.25	0.94	50.44
Rwanda	rwa	0.73	0.04	0.00	0.17
Sao Tome Principe	stp	4.88	0.44	0.12	2.00
Senegal	sen	3.40	0.20	0.04	0.94
Sierra Leone	sle	45.85	4.27	0.77	34.50
Somalia	som	1.36	0.08	0.02	0.30
North Sudan	sdn	6.58	0.07	0.02	0.36
South Sudan	ssd	18.28	0.25	0.04	2.42
Tanzania	tza	14.82	0.44	0.07	2.92
Togo	tgo	56.82	6.25	1.13	60.98
Uganda	uga	46.31	4.27	0.77	34.50
Zambia	zmb	13.29	0.44	0.07	2.92
Zimbabwe	zwe	2.01	0.07	0.02	0.30

References

1. Galactionova K, Tediosi F, Savigny Dd, Smith TA, Tanner M: **Effective coverage and systems effectiveness for malaria case management in Sub-Saharan African countries.** *PLoS ONE* 2015, **10**.
