# Supplementary material for: Distribution of malaria exposure in endemic countries in Africa considering country levels of effective treatment
Source: Malar J. 2015 Oct 5;14:384. doi: 10.1186/s12936-015-0864-3 (PMC4595196; doi:10.1186/s12936-015-0864-3)
Supplement: Supplementary file 2 — 10.1186/s12936-015-0864-3 This file includes additional Figures of results that support and expand some of the results in the main text, but whose inclusion would detract from the main argument. [file 12936_2015_864_MOESM2_ESM.pdf]

Additional File 2: Figures

Distributions of malaria exposure in endemic countries in Africa considering country levels of effective treatment

Melissa A. Penny*, Nicolas Maire, Caitlin Bever, Peter Pemberton-Ross, Olivier J.T. Briët, David L. Smith, Peter W. Gething, Thomas A. Smith

* Correspondence: melissa.penny@unibas.ch

This Additional file includes additional results that support and expand some of the results in the main text, but whose inclusion would detract from the main argument

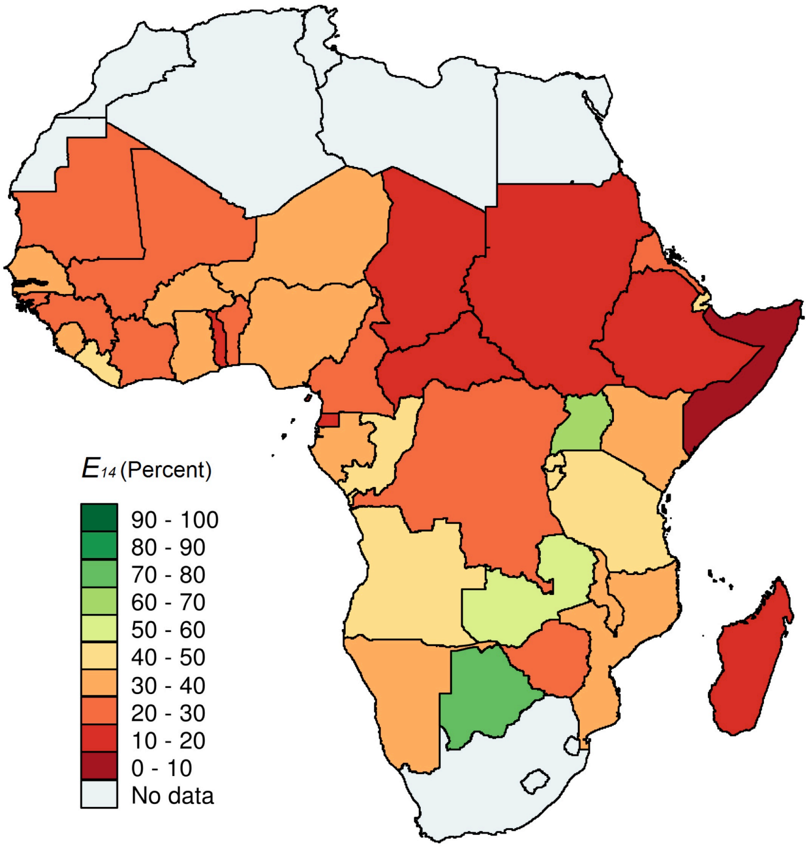

Additional File 2: Figure S1. Map of effective coverage of case management by country (values detailed in [1]).

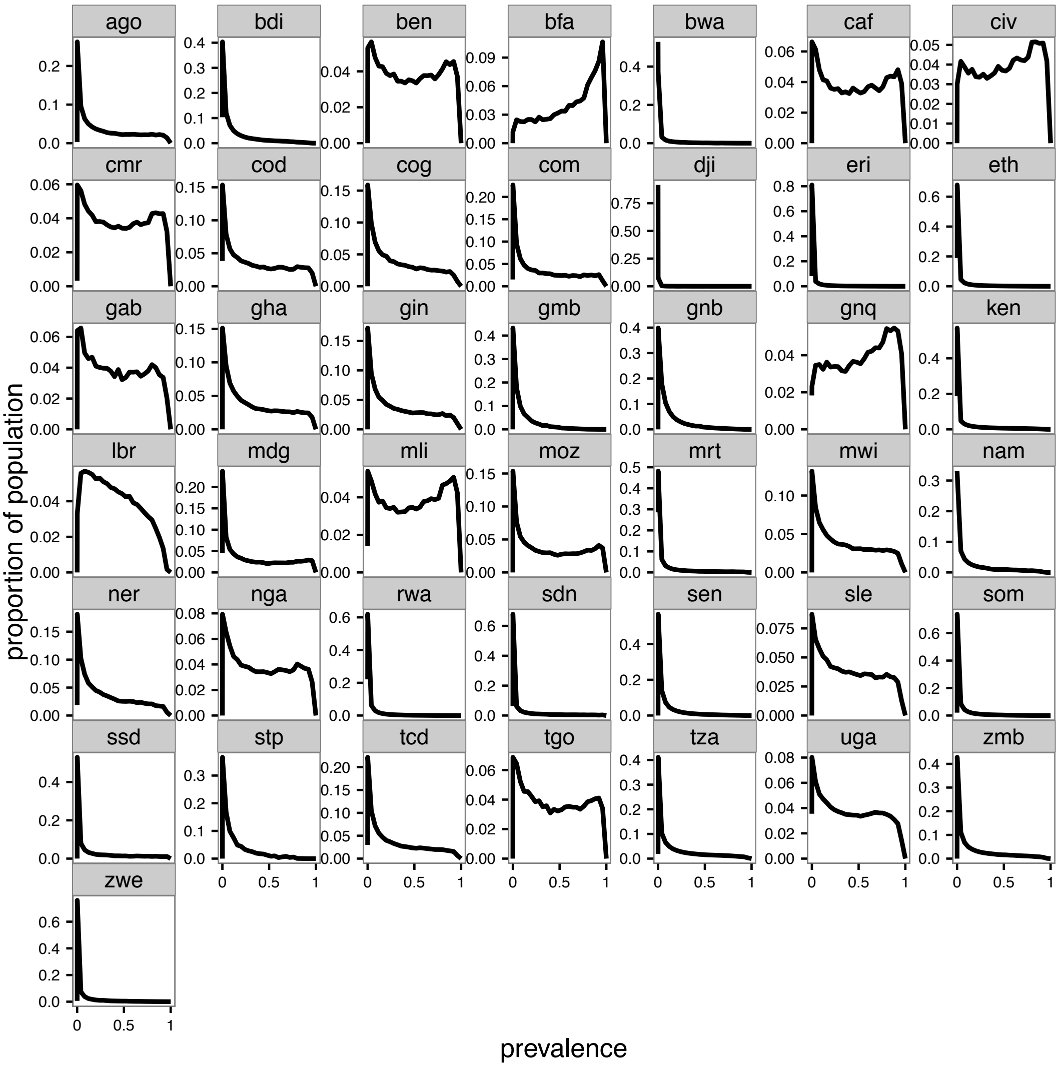

Additional File 2: Figure S2. Histograms of national level prevalence distributions

Prevalence distributions extracted from MAP, by 5km by 5km pixel and aggregated to population level. Country indicated by country code, also given in Table 2.

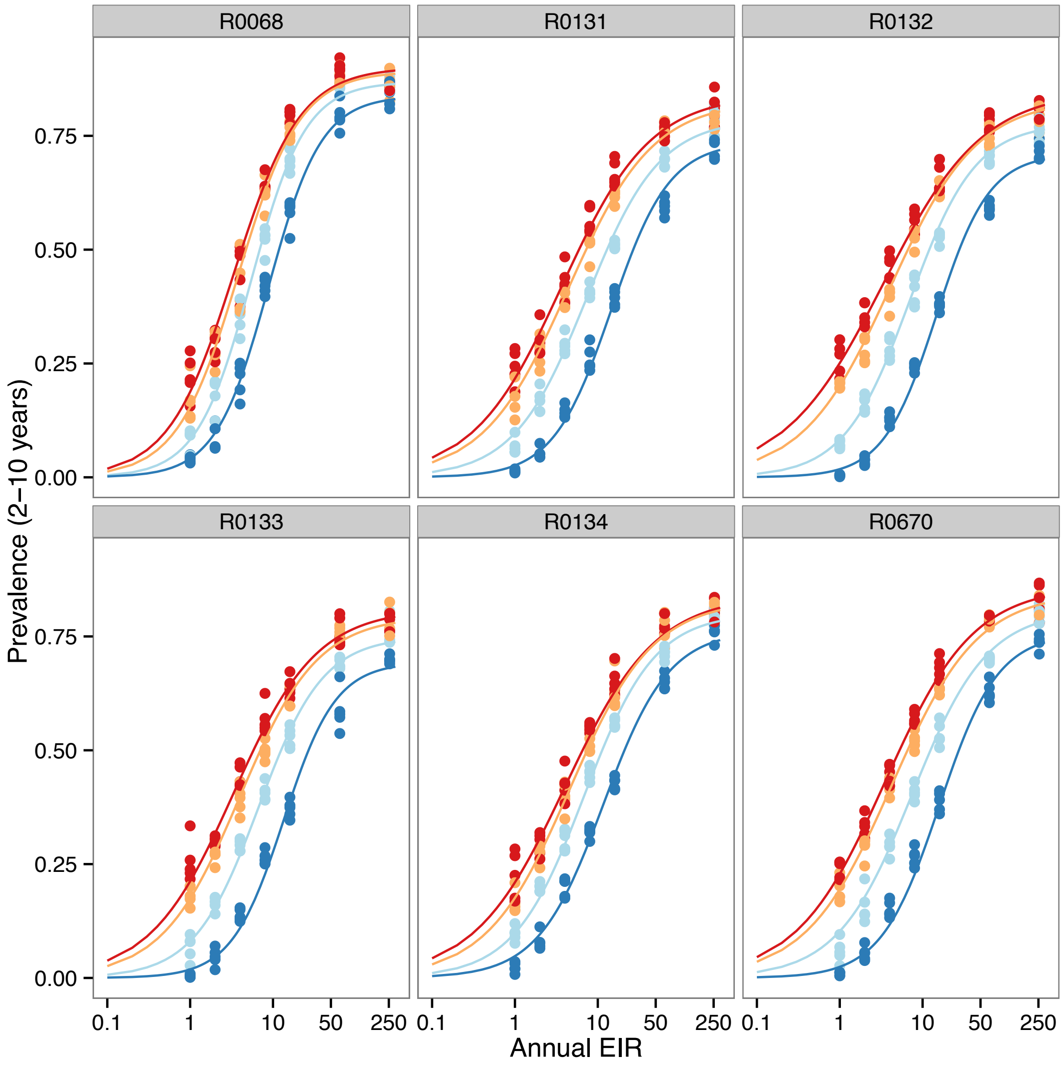

Additional File 2: Figure S3. Relationships between malaria exposure (EIR), effective coverage, and prevalence

Open Malaria model variants R0068, R0131, R0132, R0133, R0134, R0670 simulations of the relationship between prevalence and EIR for discrete levels of effective coverage (points) and best fit to these data as Hill functions (lines) (Equation (3)). Colour indicates the level of effective treatment (E_{14}), red 0.001%, yellow 5%, light blue 20%, dark blue 40%.

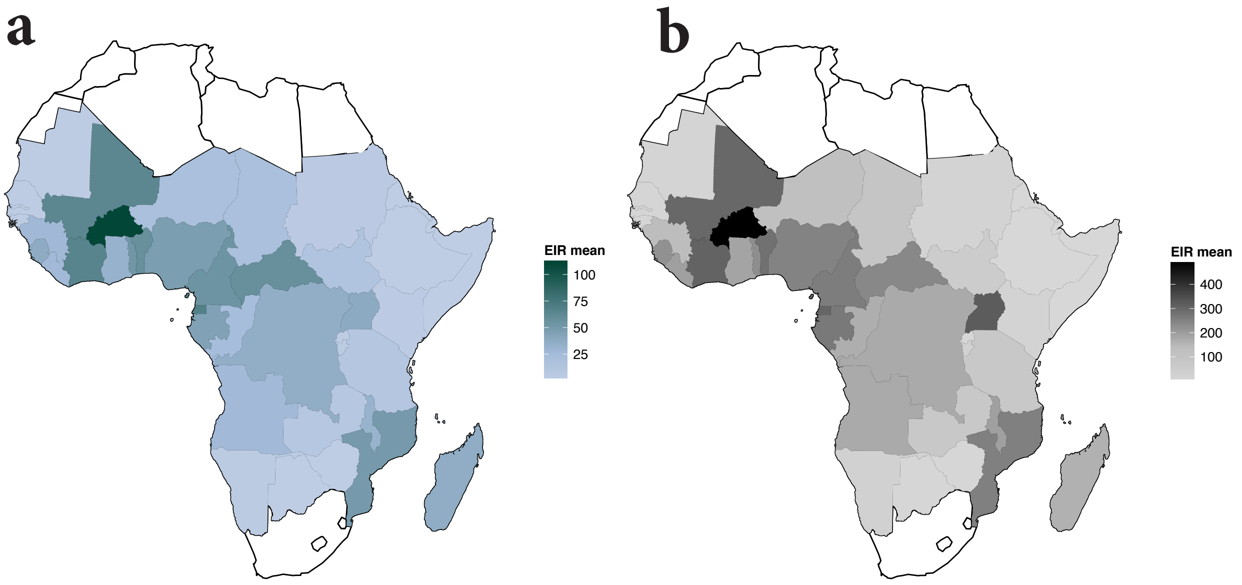

Additional File 2: Figure S4. Maps of EIR by country.

(a) Mean value of EIR (infectious bites per person per annum) calculated by method A.

(b) Mean value of EIR (infectious bites per person per annum) estimated by method B with country-specific E_{14}.

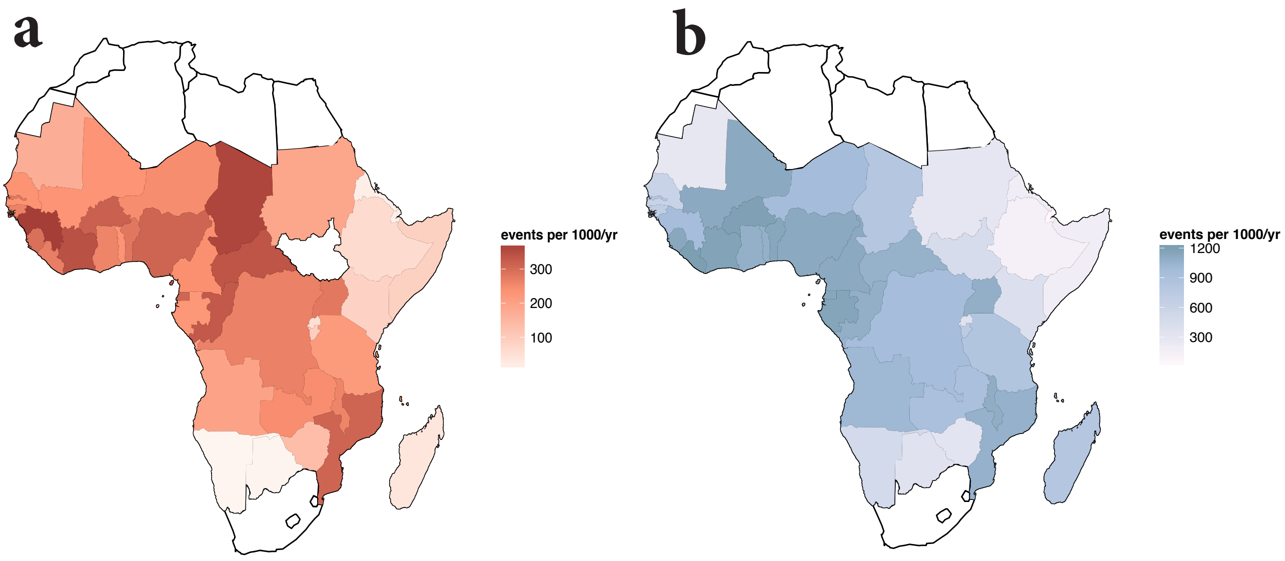

Additional File 2: Figure S5. Maps of clinical incidence by country.

(a) Incidence of clinical malaria (cases per person per annum) (WMR)

(b) Incidence of clinical malaria (episodes per person per annum) estimated by method B with country-specific E_{14}.

References

1. Galactionova K, Tediosi F, Savigny Dd, Smith TA, Tanner M: **Effective coverage and systems effectiveness for malaria case management in Sub-Saharan African countries.** *PLoS ONE* 2015, **10**.
